# Pachymic acid alleviates metabolic dysfunction-associated steatotic liver disease by inhibiting ferroptosis through PPARα

**DOI:** 10.3389/fphar.2025.1554850

**Published:** 2025-05-06

**Authors:** Guilin Ren, Yiyou Lin, Jiannan Qiu, Congcong Zhang, Lin Chen, Linwensi Zhu, Xiaohui Fan, Xiaobing Dou, Qingsheng Liu

**Affiliations:** ^1^ School of Life Sciences, Zhejiang Chinese Medical University, Hangzhou, Zhejiang, China; ^2^ Zhejiang-Hong Kong Joint Laboratory of Liver and Spleen Simultaneous Treatment in Traditional Chinese Medicine, Zhejiang Province, Hangzhou, Zhejiang, China; ^3^ Department of Gastroenterology, The First Affiliated Hospital of Zhejiang Chinese Medical University, Hangzhou, Zhejiang, China; ^4^ Innovation Center of Yangtze River Delta, Zhejiang University, Jiaxing, Zhejiang, China; ^5^ Department of Gastroenterology, Hangzhou Third People’s Hospital, Hangzhou, Zhejiang, China; ^6^ Department of Gastroenterology, Hangzhou Hospital of Traditional Chinese Medicine, Hangzhou, Zhejiang, China

**Keywords:** pachymic acid, metabolic dysfunction-associated steatotic liver disease, ferroptosis, PPARα, MAPKs signaling pathway

## Abstract

**Introduction:**

Metabolic dysfunction-associated steatotic liver disease (MASLD) is characterized by excessive lipid deposition in hepatocytes without a history of significant alcohol consumption. Pachymic acid (Pac), a bioactive triterpenoid from *Poria cocos*, has shown promise in treating MASLD due to its antioxidant and anti-inflammatory capabilities. This study aimed to elucidate the molecular impact and mechanisms of Pac in MASLD.

**Methods:**

Male C57BL/6J mice were subjected to a high-fat diet (HFD) for 8 weeks, followed by a 4 weeks treatment with the Pac. Comprehensive assessments including physiological, biochemical, and histomorphological evaluations were performed post-treatment. Transcriptomic analysis of liver samples from normal control (NC), HFD, and HFD + high-dose Pac (Pac-H) groups was conducted, with validation through Western blot, and immunofluorescence.

**Results:**

HFD induced biochemical abnormalities and liver injury, which were significantly reversed by Pac, as evidenced by decreased plasma levels of AST (Aspartate aminotransferase), ALT (Alanine aminotransferase), TG (Triglyceride), and TC (Total cholesterol), and reduced hepatic TG and TC levels. Pac also mitigated lipid accumulation, peroxidation, and ferroptosis. Pac modulated the expression of PPARα (Peroxisome proliferator-activated receptor α), MAPKs (Mitogen-activated protein kinases), and ferroptosis pathways, thereby ameliorating MASLD.

**Discussion:**

The study demonstrated that Pac inhibited the MAPKs signaling pathway and reduced hepatic ferroptosis, alleviated steatosis through PPARα regulation, offering a potential therapeutic strategy for MASLD.

## Graphical Abstract

**Figure:**
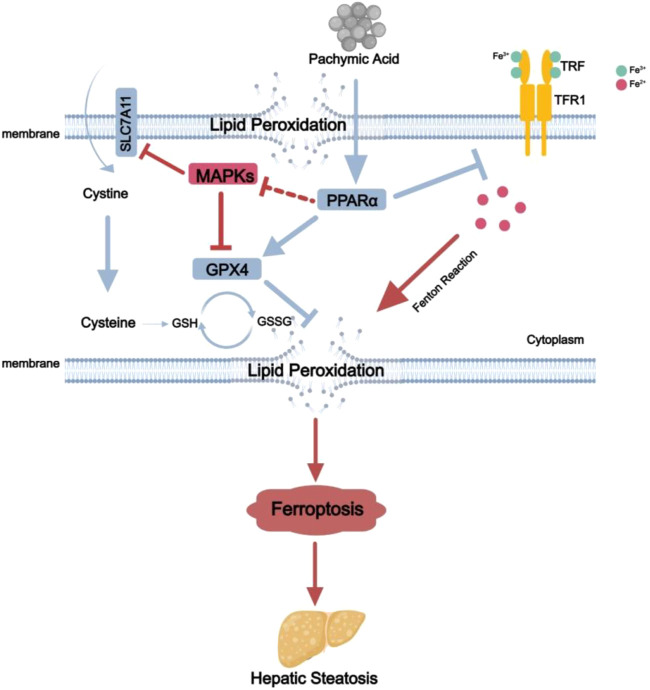
The mechanisms for Pac ameliorate non-alcoholic fatty liver disease induced ferroptosis. Pac can increase the expression of PPARα protein in the liver, which in turn upregulates GPX4 protein expression and downregulates MAPKs and TFR1 protein expression. This leads to a reduction in Fe^2+^ concentration in hepatocytes, decreases lipid peroxidation, mitigates ferroptosis, and alleviates MASLD.

## 1 Introduction

Metabolic Dysfunction-Associated Steatotic Liver Disease (MASLD), a global health concern closely linked to obesity, insulin resistance, and metabolic syndrome (Friedman et al., 2018), is characterized by abnormal lipid accumulation in hepatocytes, triggering inflammation and cellular injury. MASLD encompasses a spectrum of pathological changes, ranging from simple steatosis (metabolic dysfunction-associated steatotic liver, MASL) to metabolic dysfunction-associated steatohepatitis (MASH), marked by inflammation and hepatocyte ballooning (Byrne and Targher, 2015). Without intervention, MASLD can progress to cirrhosis and even hepatocellular carcinoma.

The complex pathophysiological mechanisms of MASLD pose significant challenges for drug development. A core driver of disease progression is oxidative stress-induced lipid peroxidation (Lin et al., 2024). Extensive studies indicate that excessive lipid accumulation in hepatocytes not only leads to cell death but also activates inflammatory cascades, driving the transition from simple steatosis to MASH.

Thus, protecting hepatocytes from death has become a critical strategy for MASLD prevention and treatment. Beyond classical forms of cell death (apoptosis, necrosis, and pyroptosis), ferroptosis—a newly recognized iron-dependent cell death pathway characterized by lipid peroxide accumulation—has gained attention. Ferroptosis disrupts iron homeostasis and lipid peroxidation, contributing to MASLD pathogenesis (Guan et al., 2023). Its regulatory network involves multiple signaling pathways, including p53, AMPK, and Keap1-Nrf2 (Xu et al., 2023; Zheng and Conrad, 2020), and offers multidimensional therapeutic targets.

Peroxisome proliferator-activated receptor alpha (PPARα), a nuclear receptor family member, shows therapeutic potential in MASLD. Studies demonstrate that PPARα agonists improve iron homeostasis, suppress inflammation, and reduce lipid peroxidation (Qu et al., 2022). PPARα has garnered significant attention for its potential therapeutic implications in MASLD. By upregulating glutathione peroxidase 4 (GPX4), PPARα enhances antioxidant defenses and provides novel insights into counteracting ferroptosis.

Pachymic acid (Pac), a triterpenoid compound derived from the medicinal fungus *Poria cocos*, exhibits anti-inflammatory, antioxidant, and anti-ferroptosis properties (Jiang et al., 2023; Ma et al., 2015). Recent findings have revealed that Pac inhibits ferroptosis in cardiomyocytes by activating the AMPK pathway, thereby alleviating myocardial ischemia/reperfusion injury (Liu et al., 2024). These characteristics suggest Pac may ameliorate MASLD through multi-target interventions.

Despite existing research on Pac’s broad bioactivities, its specific efficacy and mechanisms in MASLD remain underexplored. This study aims to systematically elucidate the hepatoprotective effects and molecular mechanisms of Pac in a high-fat diet-induced murine MASLD model, focusing on validating the hypothesis that “Pac exerts therapeutic effects in MASLD by suppressing ferroptosis through PPARα upregulation.” The findings may provide a theoretical foundation for developing natural product-based therapies for MASLD.

## 2 Materials and methods

### 2.1 Chemicals and reagents

Pachymic acid (A0575, CAS: 29070-92-6) was purchased from Chengdu must Biotechnology Co., Ltd. polyene phosphatidylcholine (H20059010) was provided by Hangzhou Traditional Chinese Medicine Hospital Center. And the high-fat diet (CAT: TP26300) was purchased from Nantong Trophic Animal Feed Co., Ltd (China), with the nutritional composition as follows: 21.2% fat, 49.1% carbohydrate, 19.8% protein, and 0.2% cholesterol.

### 2.2 Animals

The C57BL/6J male mice were provided by the Animal Experiment center of Zhejiang Chinese Medical University. After a week adaptive feeding, 40 mice were randomly divided into the NC group, the HFD group, the HFD + Pac-Low dosage group (Pac-L, 20 mg/kg), the HFD + Pac-High dosage group (Pac-H, 40 mg/kg), and the HFD + polyene phosphatidylcholine group (YSF, A positive control drug, 177.84 mg/kg) (n = 8 per group). Beginning from the eighth week of the HFD regimen, mice in the Pac-L, Pac-H received daily intragastric administration of Pac, and mice in the YSF group received daily intragastric administration of YSF for an additional 4 weeks. To investigate whether PPARα is the key target of Pac in ameliorating MASLD, another set of 32 male C57BL/6J mice were randomly divided into the NC group, the HFD group, the Pac-H group and the Pac-H + GW6471 group ((HFD + Pac-H + GW6471),GW6471, (a PPARα inhibitor; MedChemExpress, Cat: HY-15888)) (n = 8 per group). Following 8 weeks of HFD induction, the Pac-H group and the HFD + Pac-H + GW6471 group began intragastric Pac for four consecutive weeks. Concurrently, the Pac-H + GW6471 group also received intraperitoneal injections of the GW6471 for the duration of the high-fat diet induction.

All mice were housed in a temperature-controlled room maintained at 23°C–24°C with a relative humidity of 60% ± 10%, and provided with *ad libitum* access to feed and water. Their weight gain and food consumption were monitored daily. All animal experiments were conducted in accordance with the guidelines of the Institutional Animal Care and Ethics Committee of Zhejiang Chinese Medical University (Ethics Approval No. 20230424-14).

### 2.3 Bio-markers detection

Blood was collected after the mice were sacrificed and then centrifuged at 3000 *g* for 10 min at 25°C. Serum levels of alanine aminotransferase (ALT), aspartate aminotransferase (AST), triglycerides (TG), total cholesterol (TC), hepatic TG, and hepatic TC were measured using an automatic biochemical analyzer (Toshiba TK40FR, Japan). Commercial assay kits for ALT (C009-2-1), AST (C010-2-1), TG (A110-1-1), and TC (A111-1-1) were purchased from Nanjing Jiancheng Bioengineering Co., Ltd. (China).

### 2.4 Histopathology

Liver tissues were fixed via perfusion with 4% paraformaldehyde. The samples were dehydrated through a graded ethanol series (70%, 80%, 90%, and 100%) and embedded in paraffin. Subsequently, 4-μm-thick sections were prepared using a paraffin microtome and stained with hematoxylin and eosin (H&E). For Oil Red O staining, frozen tissues were embedded in Tissue-Tek OCT compound (Sakura Finetek, Torrance, CA, USA), sectioned into 8-μm-thick slices, and stained according to the manufacturer’s protocol (Solarbio, Beijing, China). Images were captured using a Carl Zeiss Axio Observer A1 inverted microscope (Oberkochen, Germany).

### 2.5 RNA preparation and sequencing

Total RNA was extracted using TRIzol reagent (Invitrogen) and quantified with a NanoDrop ND-1000. RNA integrity was verified by Agilent Bioanalyzer 2100 (RIN >7.0) and agarose gel electrophoresis (6 per group). Poly(A) RNA was enriched from 1 μg total RNA through two rounds of Dynabeads Oligo (dT)25 purification. Fragmentation was performed using the NEBNext Magnesium RNA Fragmentation Module (94°C, 5-7 min), followed by cDNA synthesis with SuperScript II Reverse Transcriptase. Second-strand cDNA was generated using *E. coli* DNA polymerase I, RNase H, and dUTP. Libraries were prepared through end-repair, A-tailing, and adapter ligation, then size-selected using AMPureXP beads. UDG-treated libraries were amplified by PCR (8 cycles) and sequenced (PE150) on an Illumina Novaseq 6000 platform.

Raw reads were processed with fastp to remove adapters and low-quality sequences. HISAT2 aligned reads to the GRCh38 human genome, followed by transcript assembly with StringTie. Merged transcriptomes were analyzed using gffcompare. Expression quantification (FPKM) and differential expression analysis were performed using StringTie and edgeR (fold change >2 or <0.5, *p* < 0.05). Final cDNA libraries showed an average insert size of 300 ± 50 bp.

### 2.6 Iron detection

The concentration of total iron (A039-2-1, Jiancheng, China), ferrous (Fe^2+^) (BC5415, Solarbio, China), and LPO (A106-1-2, Jiancheng, China) in liver tissue were detected by assay kit in accordance with manufacturer’s instructions.

### 2.7 Western blot

Liver tissues were lysed using RIPA buffer (Boster Biological Technology, Wuhan, China) supplemented with PMSF and a phosphatase inhibitor. Total protein was extracted, and protein concentration was determined using a BCA assay kit. Protein samples were loaded onto SDS-PAGE gels and transferred onto nitrocellulose membranes (Cytiva, Washington, USA). The membranes were incubated with primary antibodies overnight at 4°C, followed by incubation with corresponding secondary antibodies for 2 h at room temperature. Signals were detected using a chemiluminescence detection system, and band densities were quantified with ImageJ software (NIH, USA).

The primary antibodies used for western blotting were as follows: anti-VLDLR (rabbit, Proteintech, Cat: 19493-1-AP, 1:1000); anti-FATP5 (rabbit, Proteintech, Cat: 25347-1-AP, 1:1000); anti-β-actin (mouse, Abclonal, Cat: AC004, 1:5000); anti-Phospho-ASK1 (mouse, Santa Cruz Biotechnology, Cat: sc-166967, 1:500); anti-ASK1 (mouse, Santa Cruz Biotechnology, Cat: sc-390275, 1:500); anti-Phospho-P38 (rabbit, Cell Signaling Technology, Cat: 9211S, 1:1000); anti-Phospho-JNK (rabbit, Cell Signaling Technology, Ca: 4668S, 1:1000); anti-Phospho-ERK (rabbit, Cell Signaling Technology, Cat: 9101S, 1:1000); anti-P38 (rabbit, Cell Signaling Technology, Cat: 9212S, 1:1000); anti-JNK (rabbit, Cell Signaling Technology, Cat: 9252S, 1:1000); anti-ERK (rabbit, Cell Signaling Technology, Cat: 4695S, 1:1000); anti-Phospho-AMPK (rabbit, Cell Signaling Technology, Cat: 2535S, 1:1000); anti-AMPK (rabbit, Cell Signaling Technology, Cat: 5831S, 1:1000); anti-PPARα (mouse, Santa Cruz Biotechnology, Cat: sc-398394, 1:500); anti-SREBP1c (mouse, Santa Cruz Biotechnology, Cat: sc-365513, 1:500); anti-CD36 (mouse, Santa Cruz Biotechnology, Cat: sc-7309, 1:500); anti-SCD1 (mouse, Santa Cruz Biotechnology, Cat: sc-515875, 1:500); anti-4-HNE (mouse, Thermo Fisher Scientific, Cat: MA5-27570, 1:1000); anti-TFR1 (rabbit, AiFang Biological, Cat: AF03632, 1:500); anti-GPX4 (mouse, Proteintech, Cat: 67763-1-IG, 1:1000); anti-SLC7A11 (rabbit, Cell Signaling Technology, Cat: 98051S, 1:1000). Secondary antibodies included: anti-mouse IgG-HRP (Boster, Cat: BA1054, 1:5000); anti-rabbit IgG-HRP (Boster, Cat: BA1050, 1:5000).

### 2.8 Immunofluorescence staining

Liver tissues were incubated with anti-TFR1 (Santa Cruz Biotechnology, Cat: SC-393719, 1:500) at 4°C for 12 h to assess hepatic Fe^2+^ uptake. After washing, the sections were stained with AF488-conjugated goat anti-rabbit IgG (H + L) (Dawen Biotec, Cat: WD-GAR4881, 1:500) at room temperature for 1 h. Finally, the sections were imaged using a digital slide scanner (Olympus VS200, Tokyo, Japan) and analyzed with OlyVIA 4.1.1 software.

### 2.9 Mitochondrial function and morphology detection in AML-12 cells

AML-12 cells in the logarithmic growth phase were seeded into confocal dishes at a density of 3 × 10^4^ cells/dish. After 12 h of incubation, cells were pretreated with drugs for 2 h, followed by the addition of PO (BSA-conjugated palmitate:oleate complex, 2:1 ratio; Sigma-Aldrich, St. Louis, MO, USA) to establish the MASLD model, and further cultured for 12 h. The supernatant was discarded, and cells were washed twice with PBS. Subsequently, cells were incubated for 1 h in medium containing MitoTracker Deep Red 633 (Beyotime, Cat: C1034, 1:1000), BODIPY 493/503 (Proteintech, Cat: CM02294, 1:1000), and Hoechst 33342 (Beyotime, Cat: C1028, 1:1000). After staining, the supernatant was removed, and cells were washed twice with PBS before fresh medium was added. Finally, images were acquired using a confocal laser scanning microscope (LSM 900, Carl Zeiss AG, Jena, Germany) and analyzed with ZEN 2012 software.

### 2.10 Data analysis

The data were presented as the mean ± standard deviation (SD). Multi-group comparisons were performed using one-way ANOVA followed by a *post hoc* test with Fisher’s least significant difference, conducted in GraphPad Prism nine software (Graphpad Software, Inc; San Diego, CA, USA). Comparison between two groups was analyzed using Student’s t-test. Statistical significance was defined as a *p*-value <0.05.

## 3 Results

### 3.1 Pac alleviated hepatic steatosis in MASLD mice

**FIGURE 1:**
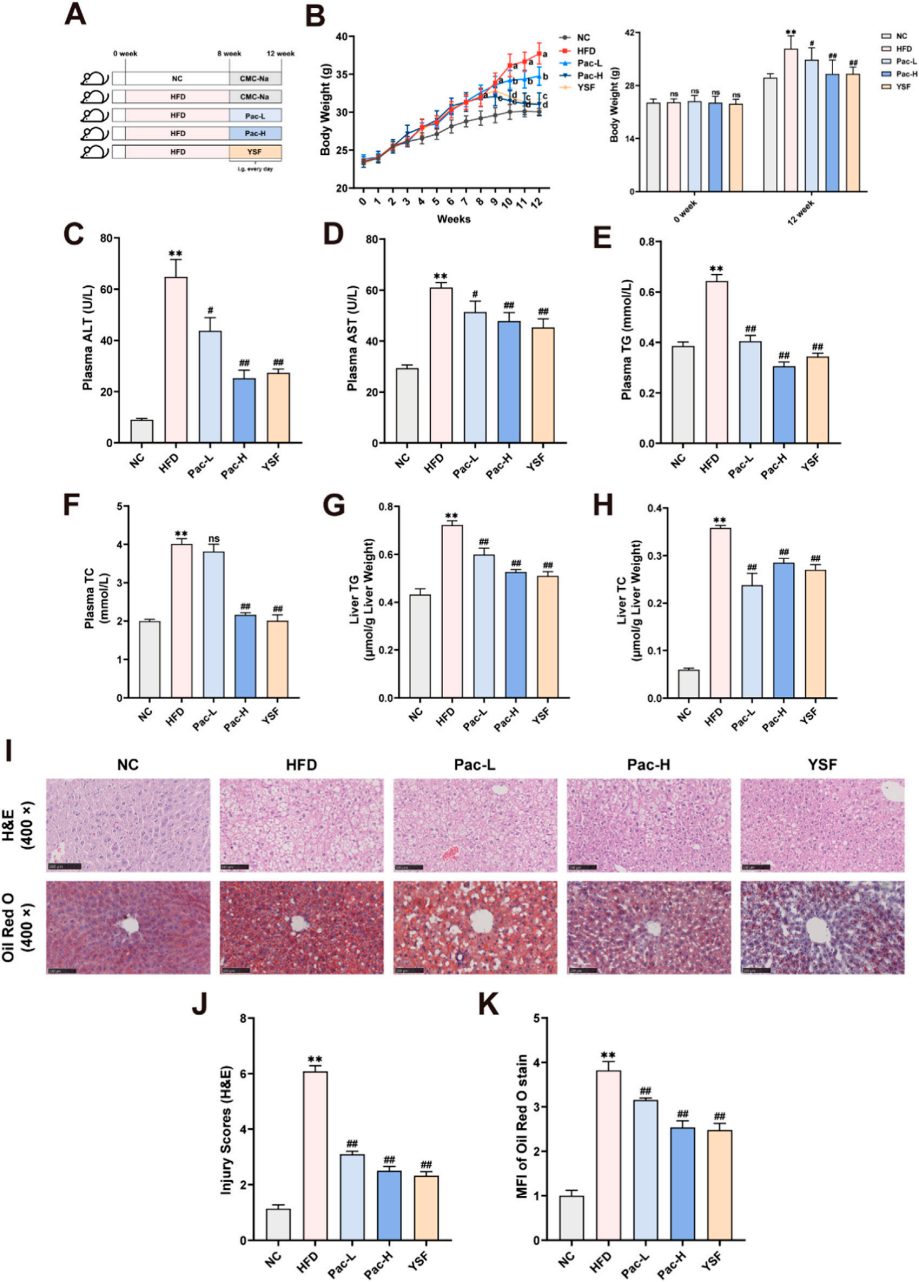
Pac effectively reduced hepatic steatosis and injury in MASLD mice. **(A)** Experimental flowchart. **(B)** The wight growth curve of each group (weekly). **(C,D)** The level of plasma ALT and AST. **(E,F)** The level of plasma TG and TC. **(G,H)** Hepatic lipid (TG and TC) levels. **(I–K)** Representative images of liver tissues stained with H&E (scale bar = 100 μm) or Oil Red O (scale bar = 100 μm). Data are showed as mean ± SD (n = 4–8). *p value for v. s. NC < 0.05, **p value for v. s. NC < 0.01, ^#^ *p* value for v. s. HFD <0.05, ^##^ *p* value for v. s. HFD <0.01.

The therapeutic effect of Pac on hepatic steatosis was evaluated in HFD-induced MASLD mouse model. MASLD was induced by 12-week HFD feeding, after which Pac and polyene phosphatidylcholine (YSF, a positive control drug) were administered intragastrically daily from the eighth week for 4 weeks (Figure 1A). Pac and YSF treatments significantly reduced body weight in HFD-fed mice compared to the HFD control group (Figure 1B). Plasma levels of AST and ALT in Pac-treated MASLD mice were significantly lower than those in the HFD group (Figures 1C,D). Compared to the normal control (NC) group, the HFD group exhibited markedly elevated levels of TC and TG in both liver and serum, which were effectively attenuated by Pac treatment (Figures 1E–H). Oil Red O staining revealed substantial lipid droplet accumulation in the liver tissue after 12 weeks of HFD feeding (Figures 1I–K). Histopathological analysis further demonstrated disrupted hepatic lobular architecture and pronounced inflammatory cell infiltration in the HFD group, both of which were ameliorated by Pac treatment. Notably, the therapeutic efficacy of Pac was comparable to that of YSF.

### 3.2 The PPAR and MAPK signaling pathways contribute to the ferroptosis of MASLD following Pac therapy

**FIGURE 2:**
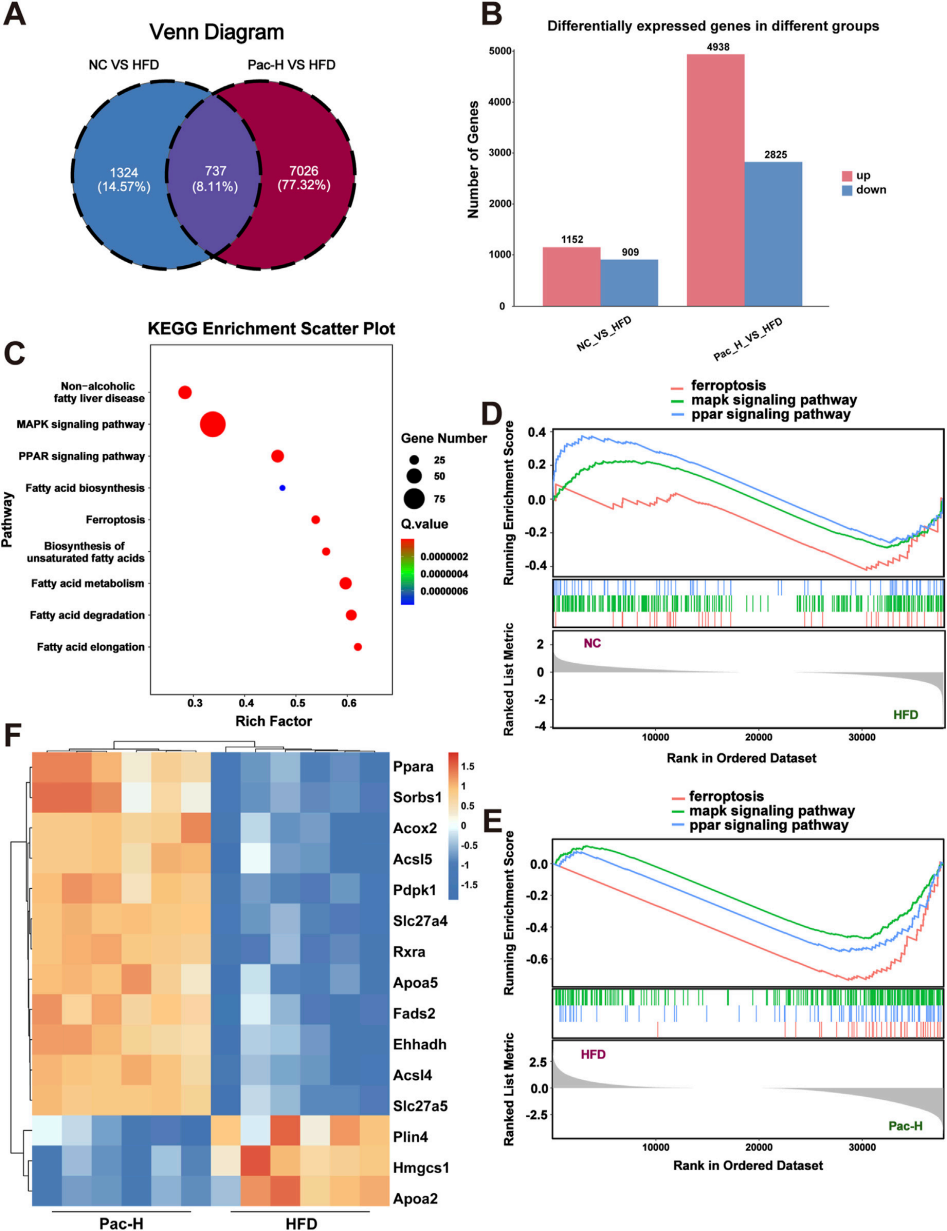
RNA sequence analysis used to explore the mechanism of Pac on MASLD. **(A)** Veen diagram of DEGs among each group. **(B)** KEGG enrichment analysis based on differential genes. **(C)** Differentially expressed genes between the NC group and the HFD group and between the HFD group and the Pac group, respectively. **(D,E)** The Gene Set Enrichment Analysis (GSEA) of ferroptosis, MAPKs signaling pathway and PPAR signaling pathway in each group. **(F)** The heatmap of Differential genes. n = 6 per group.

To delineate the transcriptomic landscape and identify molecular targets underlying Pac’s therapeutic effects, we performed RNA sequencing on liver tissues from the NC, HFD, and HFD + Pac groups. Venn diagram analysis revealed 737 genes with overlapping expression across all three groups (Figure 2A). Compared to the NC group, the HFD group exhibited 1,152 upregulated and 909 downregulated genes. In contrast, Pac treatment reversed this trend, with 4,938 upregulated and 2,825 downregulated genes relative to the HFD group (Figure 2B). KEGG pathway enrichment analysis highlighted significant associations with the MAPK Signaling Pathway, PPAR Signaling Pathway, and Ferroptosis-related genes (Figure 2C).

Gene Set Enrichment Analysis (GSEA) using Pearson’s correlation showed that the HFD group had negative enrichment in the PPAR Signaling Pathway, MAPK Signaling Pathway, and Ferroptosis, whereas Pac treatment positively regulated these pathways (Figures 2D,E). These findings suggested that Pac’s anti-MASLD effects were mediated through modulation of these pathways.

The heatmap further demonstrated PPARα as a key mediator of Pac’s efficacy (Figure 2F). Collectively, these data implicate the PPAR Signaling Pathway, MAPK Signaling Pathway, and Ferroptosis in Pac’s therapeutic mechanism. To validate this, we employed GW6471 in subsequent experiments.

### 3.3 Pac alleviated lipid metabolism disorder in MASLD mice

**FIGURE 3:**
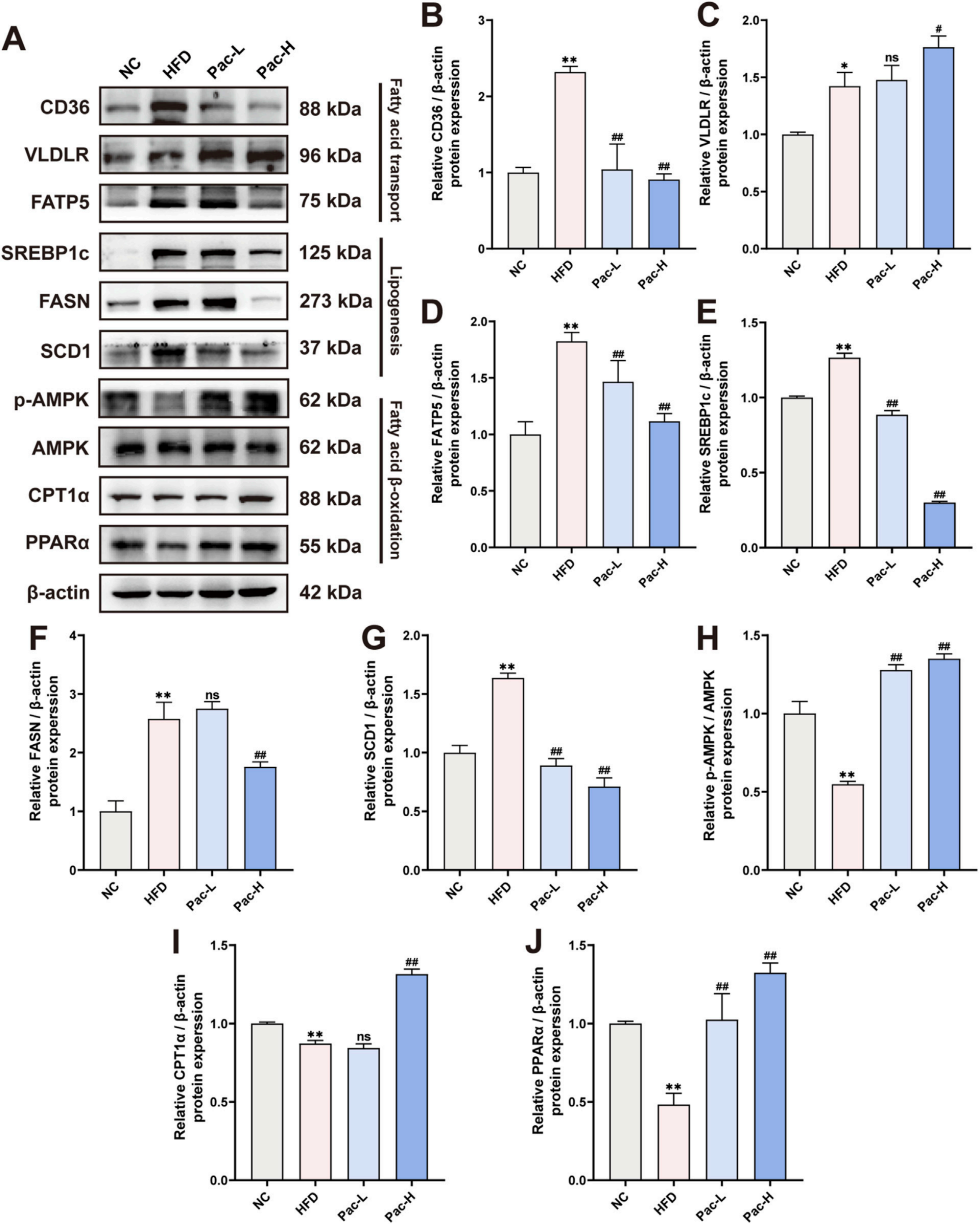
Pac protected HFD-induced MASLD in mice by improving lipid metabolism. **(A–J)** The protein levels of CD36, VLDLR, FATP5, SREBP1c, FASN, SCD1, p-AMPK, AMPK, CPT1α, PPARα in mice liver were detected by Western blot. Data are showed as mean ± SD (n = 3). *p value for v. s. NC < 0.05, **p value for v. s. NC < 0.01, ^#^ *p* value for v. s. HFD <0.05, ^##^ *p* value for v. s. HFD <0.01, ns means no significant.

The aberrant regulation of lipid metabolism constitutes a central pathophysiological feature in the transition from MASLD to MASH. To elucidate the underlying mechanisms, we quantified the protein expression of key enzymes involved in fatty acid transport (including CD36, VLDLR, and FATP5), lipogenesis (SREBP1c and FASN), and fatty acid β-oxidation (p-AMPK, AMPK, CPT1α, and PPARα) through Western blot analysis of MASLD mouse models. As shown in Figures 3A–J, Pac treatment markedly reduced the hepatic protein levels of CD36 and FATP5. Similarly, Pac administration led to decreased expression of lipogenesis-associated proteins, SREBP1c and FASN. Notably, Pac not only upregulated PPARα and CPT1α but also enhanced AMPK phosphorylation, suggesting its dual role in stimulating fatty acid β-oxidation. These observations collectively highlight the efficacy of Pac in alleviating MASLD through modulating lipid synthesis, enhancing fatty acid transport, and promoting fatty acid β-oxidation. Our findings demonstrate that Pac administration significantly mitigates hepatic lipid metabolic dysfunction in MASLD mice.

### 3.4 Pac improved hepatic ferroptosis in MASLD mice

**FIGURE 4:**
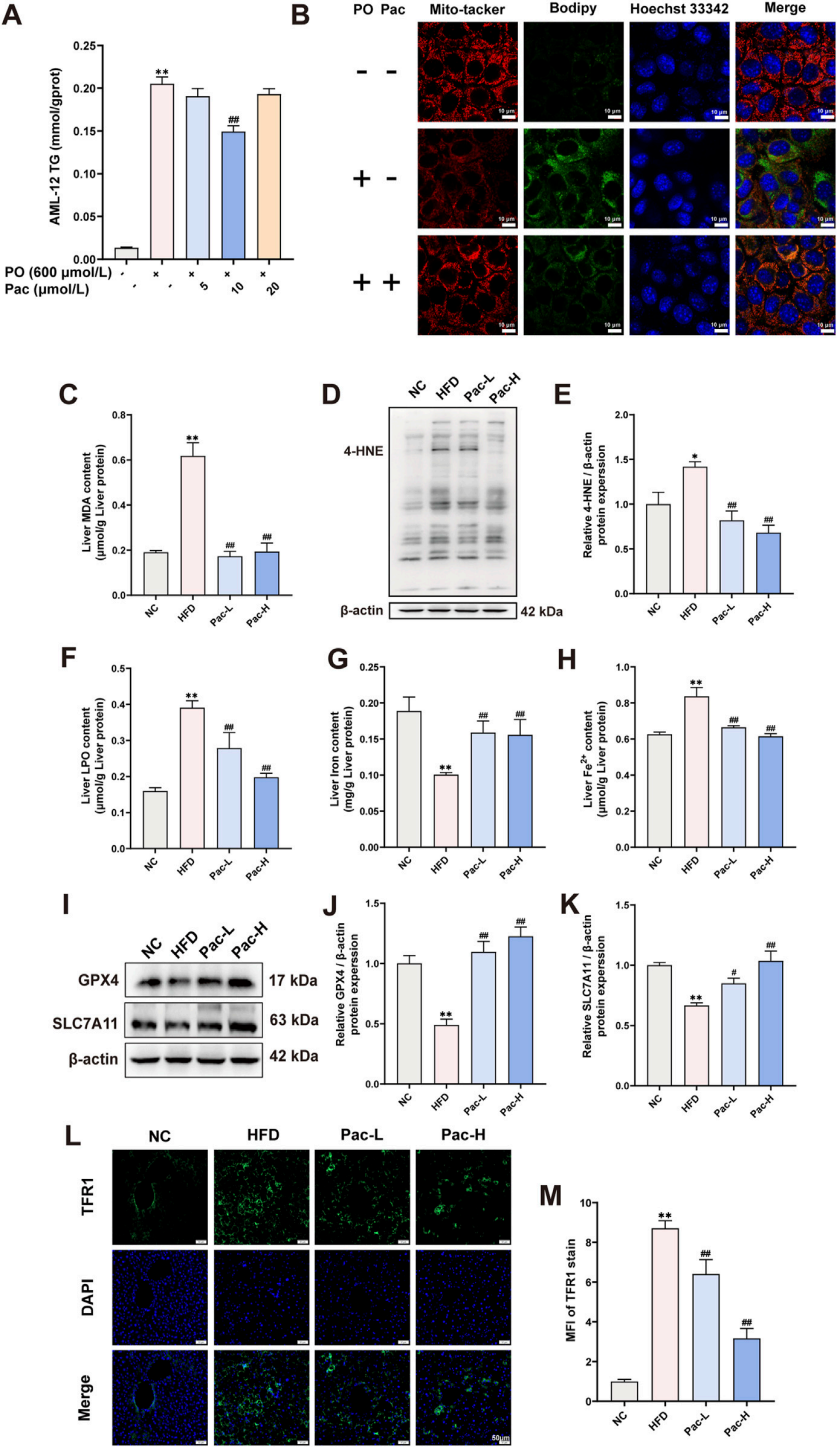
Pac improved hepatic ferroptosis in MASLD mice. **(A)** The level of TG in the AML-12 cells. **(B)** Representative images of liver tissues stained with Mito-tracker, Bodipy, and Hoechst33342 (scale bar = 10 μm). **(C)** The level of MDA in the liver. **(D ,E)** The liver level of 4-HNE accessed by Western Blot. **(F)** The level LPO content in the liver of mice. **(G,H)** The level iron content, and the levels of Fe^2+^ content in the liver of mice. Data are showed as mean ± SD (n = 4–8). **(I–K)** The protein levels of GPX4, SLC7A11 in MASLD mice. **(L, M)** Immunofluorescence assay on the protein expression of TFR1. Data are showed as mean ± SD (n = 3). *p value for v. s. NC < 0.05, **p value for v. s. NC < 0.01, #*p* value for v. s. HFD <0.05, ##p value for v. s. HFD <0.01, ^△^ *p* value for v. s. Pac <0.05, ^△△^ *p* value for v. s. Pac <0.01.

Based on transcriptomic analysis, we established a MASLD model in AML-12 cells using PO (600 μmol/L). Following Pac intervention, we observed that 10 μmol/L Pac significantly reduced intracellular accumulation of PO-induced triglycerides (Figure 4A). In subsequent experiments, Pac administration was consistently maintained at 10 μmol/L.

Mitochondrial morphological and functional abnormalities are key features of ferroptosis. Mito-Tracker staining revealed that PO treatment induced mitochondrial fragmentation in AML-12 cells, which was significantly reversed by Pac (Figure 4B). Bodipy staining further demonstrated that Pac reduced intracellular lipid droplet accumulation (Figure 4C). In HFD-induced mice, hepatic malondialdehyde (MDA) content was markedly elevated, and Pac treatment significantly attenuated this increase (Figure 4D). Similarly, Pac administration downregulated the expression of 4-hydroxynonenal (4-HNE), another oxidative stress marker, compared to the HFD group (Figures 4E,F). Pac also elevated liver iron content while decreasing ferrous iron (Fe^2+^) levels and lipid peroxidation (LPO) in MASLD mice (Figures 4G–I).

Western blot analyses confirmed that Pac increased the expression of glutathione peroxidase 4 (GPX4) and solute carrier family seven member 11 (SLC7A11) in MASLD mouse liver tissue (Figures 4J–L). Notably, Pac upregulated transferrin receptor 1 (TFR1) protein expression (Figures 4M,N). Collectively, these data demonstrate that Pac protects against hepatic ferroptosis in MASLD by modulating oxidative stress and iron metabolism, consistent with RNA sequencing findings. This highlights Pac’s potential as a therapeutic agent for MASLD-related hepatic oxidative stress.

### 3.5 Pac ameliorate MASLD mice by inhibiting MAPKs signal pathway

**FIGURE 5:**
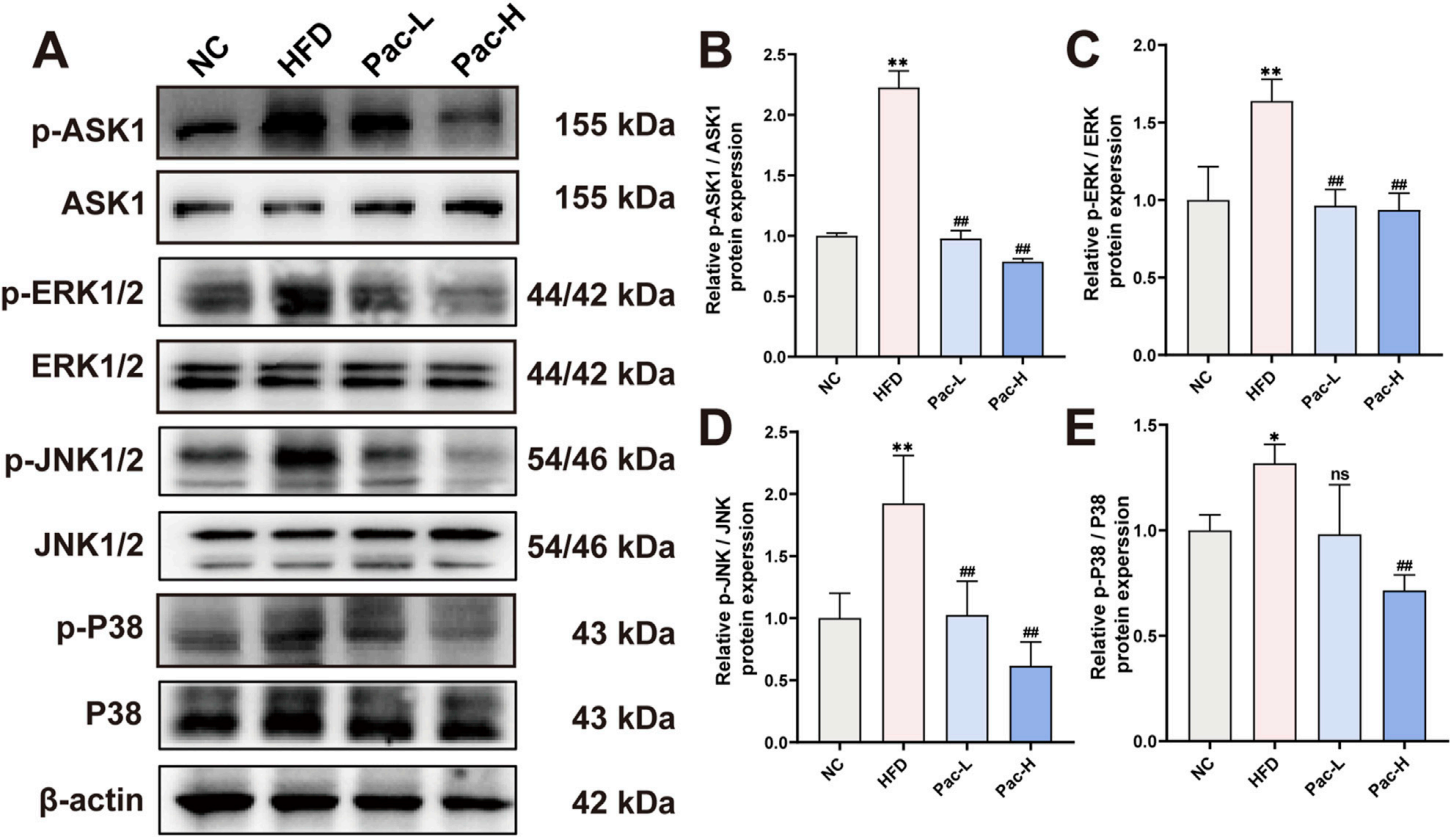
Pac ameliorate MASLD though MAPKs signal pathway **(A–E)** Protein level of p-ASK1, ASK, 1 p-ERK, ERK, p-JNK, JNK, p-P38, P38 in MASLD mice. Data are showed as mean ± SD (n = 3). *p value for v. s. NC < 0.05, **p value for v. s. NC < 0.01, ^**#**^ *p* value for v. s. HFD <0.05, ^**##**^ *p* value for v. s. HFD <0.01, ns means no significant.

To substantiate the hypothesis that Pac alleviates MASLD by modulating the MAPK signaling pathway, we performed Western blot analyses. Our findings revealed a pronounced upregulation of phosphorylated ASK (p-ASK), ERK (p-ERK), JNK (p-JNK), and P38 (p-P38) in the liver tissues of MASLD mice. Notably, Pac treatment significantly reversed the elevated expression of these phosphorylated MAPK components (Figures 5A–E). These results collectively suggest that Pac mitigates MASLD by inhibiting the MAPK signaling pathway in mice.

### 3.6 Hepatic lipid accumulation suppressed when PPARα was inhibited

**FIGURE 6:**
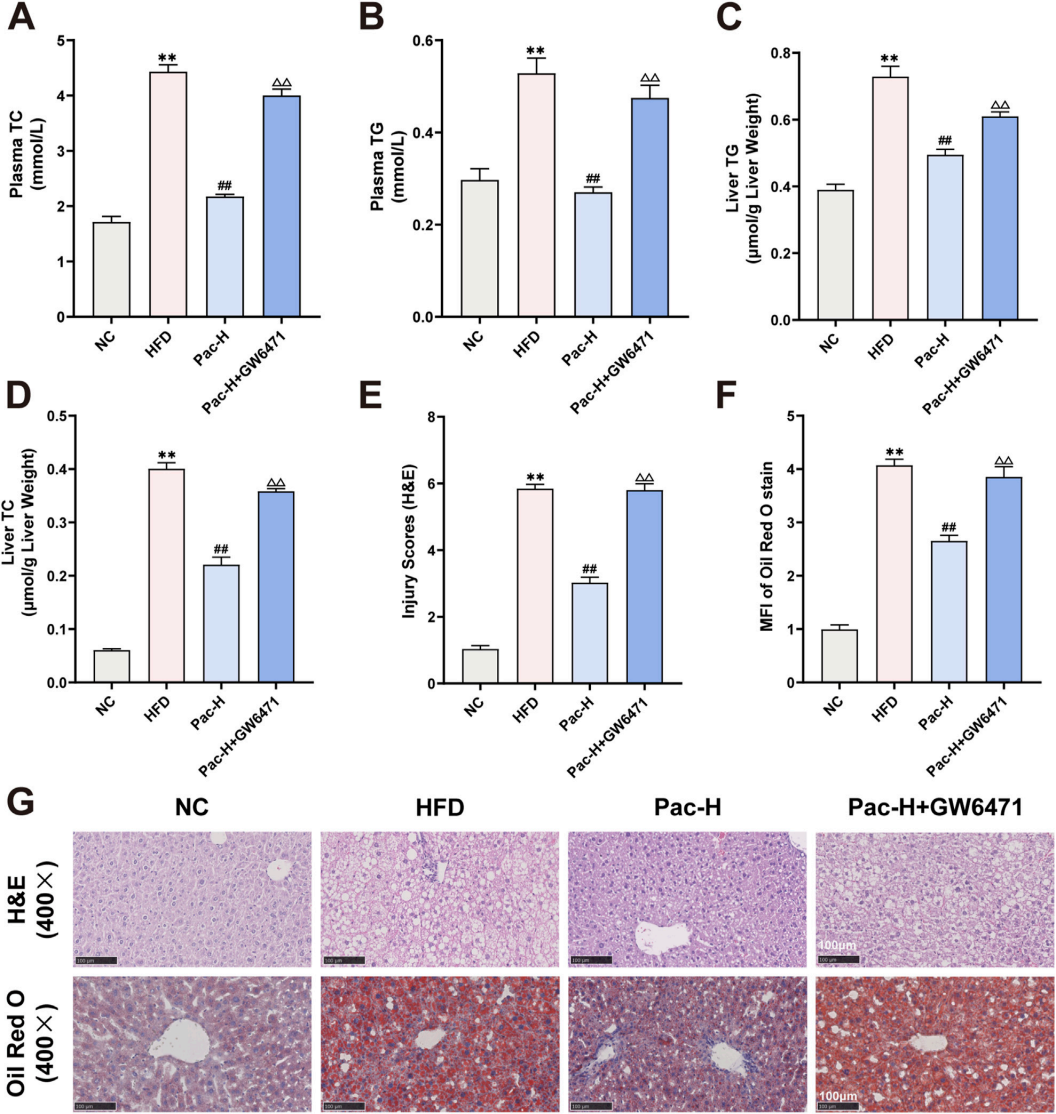
GW6471 abolished the anti-lipid accumulation effect of Pac in MASLD mice. **(A)** The plasma level of TC. **(B)** The plasma level of TG. **(C)** The level of TG in the liver. **(D)** The level of TC in the liver. **(E–G)** Representative images of liver tissues stained with H&E (scale bar = 100 μm) or Oil Red O (scale bar = 100 μm). Data are showed as mean ± SD (n = 4–8). *p value for v. s. NC < 0.05, **p value for v. s. NC < 0.01, ^**#**^ *p* value for v. s. HFD <0.05, ^**##**^ *p* value for v. s. HFD <0.01, ^△^ *p* value for v. s. Pac <0.05, ^△△^ *p* value for v. s. Pac <0.01.

To determine whether PPARα is essential for the therapeutic effects of Pac against MASLD, we administered the PPARα antagonist GW6471 (Supplementary Figure S1A). GW6471 markedly attenuated Pac-induced body weight reduction in HFD-fed mice (Supplementary Figure S1B). Pac significantly decreased serum levels of AST, ALT, TC, and TG, as well as hepatic TC and TG content. However, co-treatment with GW6471 abolished these beneficial effects (Supplementary Figure S1C, D; Figure 6A-D). Histopathological analysis further showed that GW6471 completely blocked Pac’s hepatoprotective effects, including restoration of liver tissue integrity and reduction of lipid accumulation in MASLD mice (Figures 6E–G).

**FIGURE 7:**
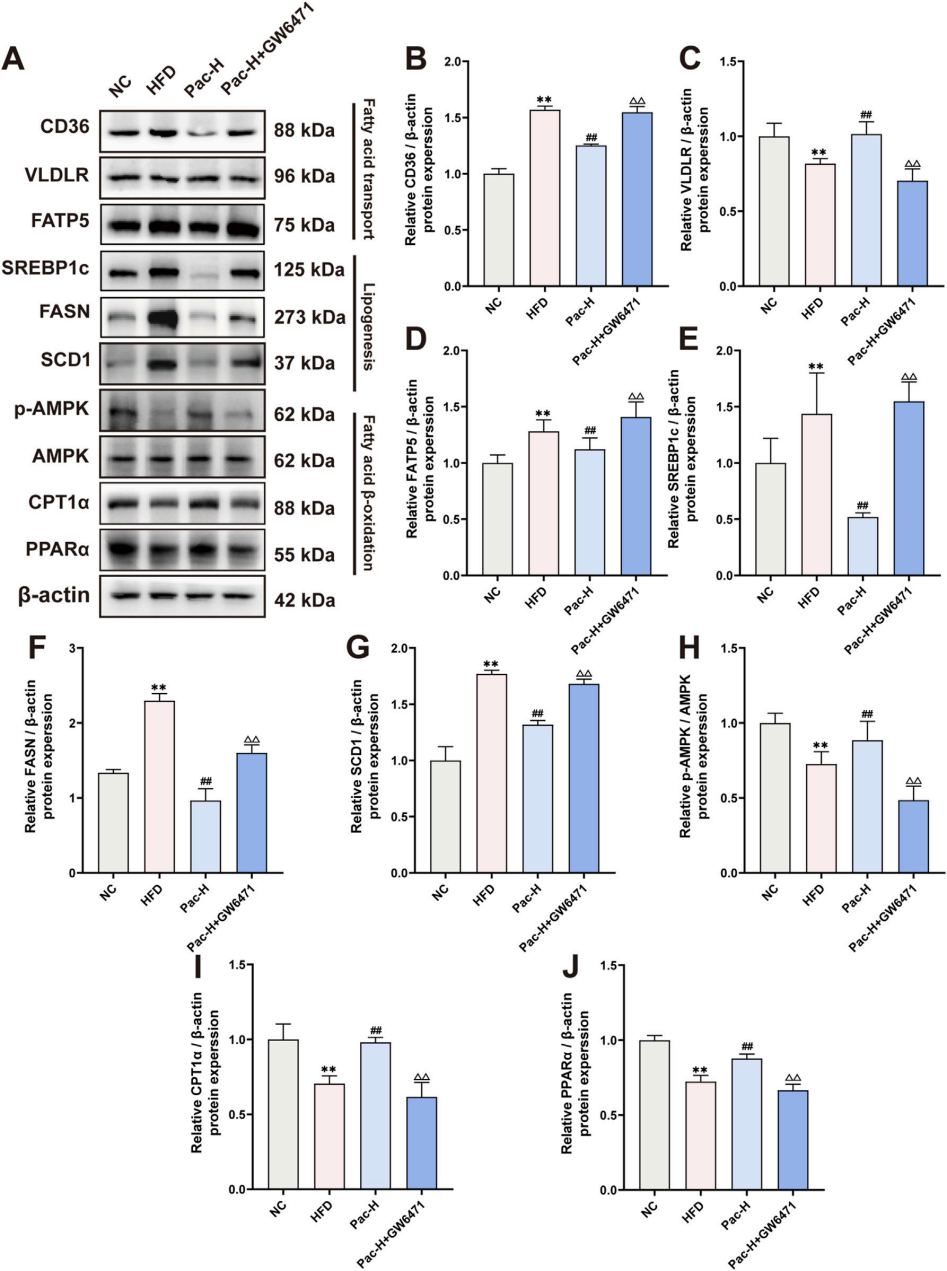
GW6471 abolished the effect on lipid metabolism of Pac in NAFLD mice. **(A–J)** The protein levels of CD36, VLDLR, FATP5, SREBP1c, FASN, SCD1, p-AMPK, AMPK, CPT1α, PPARα in mice liver were detected by Western blot. Data are showed as mean ± SD (n = 3). *p value for v. s. NC < 0.05, **p value for v. s. NC < 0.01, ^**#**^ *p* value for v. s. HFD <0.05, ^**##**^ *p* value for v. s. HFD <0.01, ^△^ *p* value for v. s. Pac <0.05, ^△△^ *p* value for v. s. Pac <0.01.

We also examined GW6471s impact on lipid metabolism and inflammatory gene expression. Pac modulated the expression of lipid metabolism- and inflammation-related proteins in MASLD mice, but GW6471 abolished these regulatory effects (Figures 7A–J). These findings indicate that Pac’s beneficial effects on lipid metabolism in MASLD mice depend on PPARα activity, underscoring PPARα′s pivotal role in mediating Pac’s therapeutic efficacy.

### 3.7 The Pac’s ability to improve liver ferroptosis was suppressed by PPARα inhibition

**FIGURE 8:**
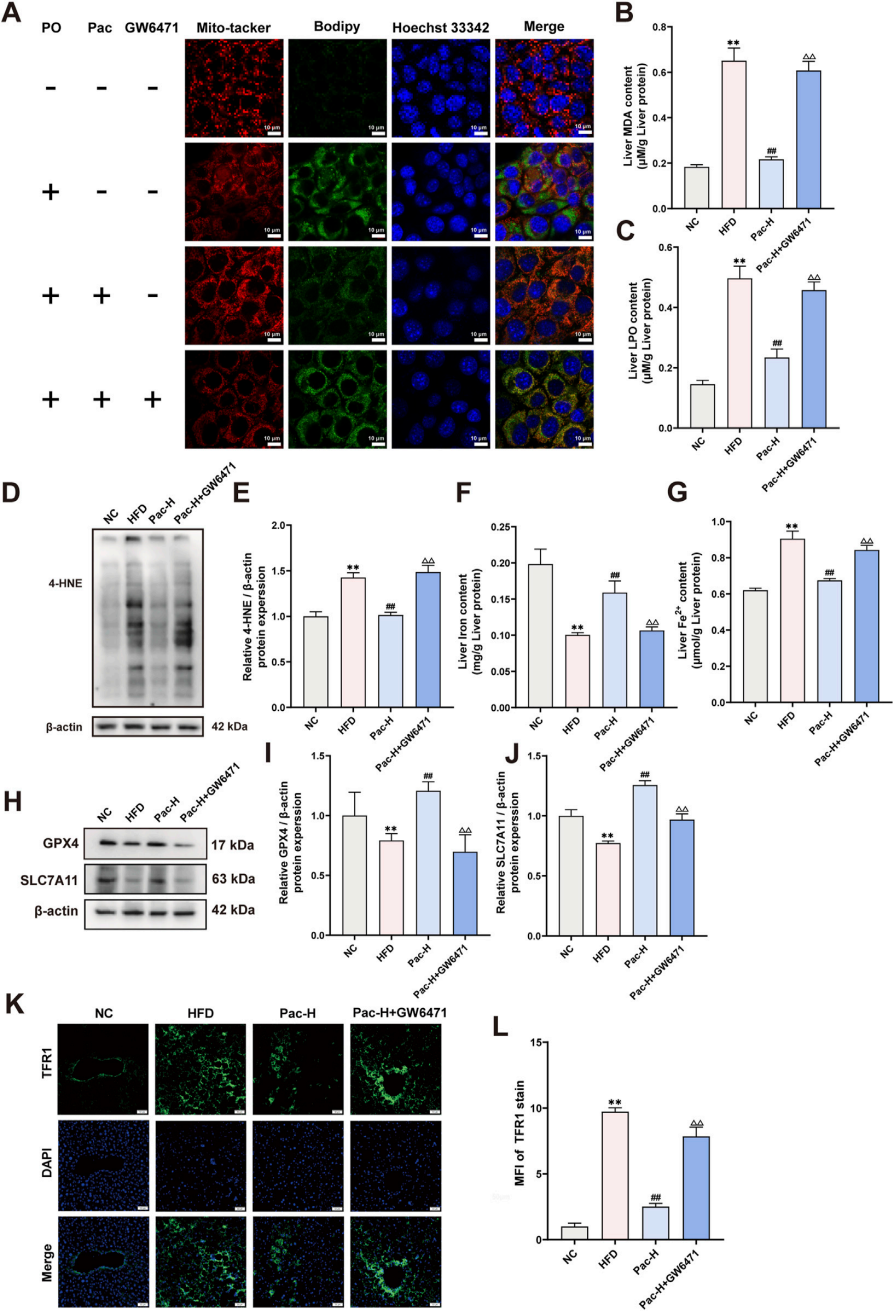
GW6471 abolished the anti-hepatic ferroptosis effect of Pac in MASLD mice. **(A)** Representative images of liver tissues stained with Mito-tracker, Bodipy, and Hoechst33342 (scale bar = 10 μm). **(B)** The level of MDA in the liver. **(C)** The level LPO content in the liver of mice. **(D,E)** The liver level of 4-HNE accessed by Western Blot. **(F,G)** The level iron content, and the levels of Fe^2+^ content in the liver of mice. Data are showed as mean ± SD (n = 4–8). **(H,J)** The protein levels of GPX4, SLC7A11 in MASLD mice. **(K, L)** Immunofluorescence assay on the protein expression of TFR1. Data are showed as mean ± SD (n = 3). *p value for v. s. NC < 0.05, **p value for v. s. NC < 0.01, ^#^ *p* value for v. s. HFD <0.05, ^##^ *p* value for v. s. HFD <0.01, ^△^ *p* value for v. s. Pac <0.05, ^△△^ *p* value for v. s. Pac <0.01.

To further investigate the mechanism by which Pac inhibits hepatic ferroptosis via PPARα in MASLD mice, we administered the PPARα inhibitor GW6471. GW6471 attenuated Pac’s ability to reduce triglyceride levels in AML-12 cells (Supplementary Figure S1E). Mito-Tracker and Bodipy staining showed that GW6471 diminished Pac-induced improvements in mitochondrial morphology (Figure 8A). Additionally, GW6471 reversed Pac-mediated reductions in hepatic LPO and MDA content in MASLD mice (Figures 8B,C). Pac also inhibited hepatic iron ion accumulation, an effect significantly blunted by GW6471 (Figures 8F,G). Western blot analysis demonstrated that Pac upregulated GPX4 and SLC7A11, both critical for maintaining redox homeostasis and suppressing ferroptosis. However, GW6471 abolished these effects (Figures 8H–J). Conversely, Pac plus GW6471 increased the expression of 4-HNE (a lipid peroxidation marker) and TFR1 (an iron uptake regulator) compared to Pac alone (Figures 8D,E,K,L). These results strongly suggest that Pac protects against ferroptosis by activating PPARα, highlighting PPARα′s essential role in mediating Pac’s anti-ferroptotic effects in MASLD.

### 3.8 The inhibition of Pac on MAPKs signal pathway was weakened by PPARα inhibition

To investigate whether Pac modulates the MAPK signaling pathway in MASLD via PPARα activation, we used the selective PPARα inhibitor GW6471. Notably, our data clarify a key discrepancy: while initial reports suggested Pac increases phosphorylation of ASK1 (p-ASK1), ERK (p-ERK), JNK (p-JNK), and P38 (p-P38), it is now established that Pac suppresses their phosphorylation, thereby inhibiting MAPK signaling.

**FIGURE 9:**
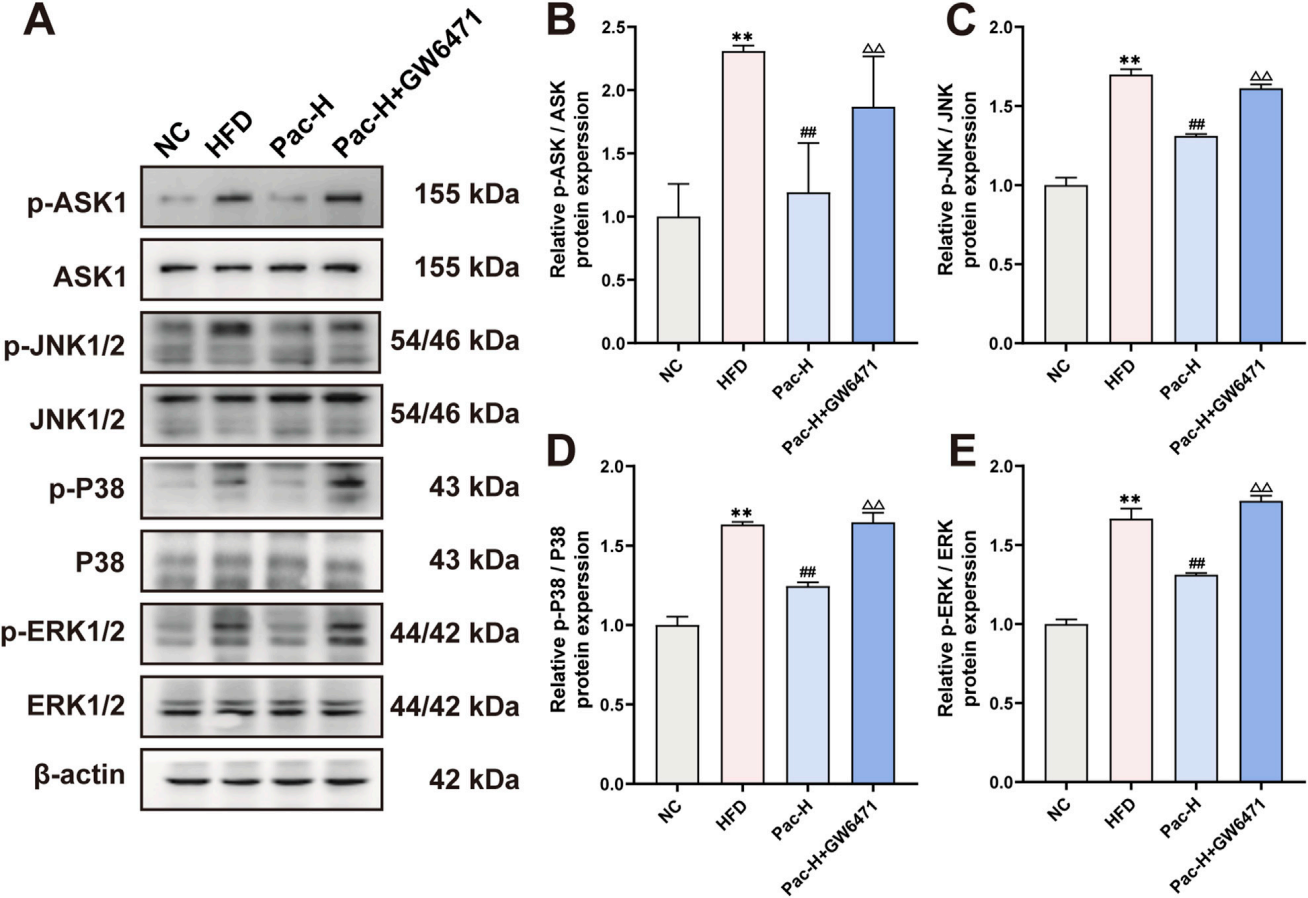
GW6471 abolished the inhibited effect of Pac on MAPKs pathway in MASLD mice. **(A–E)** Protein level of p-ASK, ASK, p-ERK, ERK, p-JNK, JNK, p-P38, P38 in MASLD mice. Data are shown as mean ± SD (n = 3). *p value for v. s. NC < 0.05, **p value for v. s. NC < 0.01, ^**#**^ *p* value for v. s. HFD <0.05, ^**##**^ *p* value for v. s. HFD <0.01, ^△^ *p* value for v. s. Pac <0.05, ^△△^ *p* value for v. s. Pac <0.01.

Co-treatment with GW6471 attenuated Pac’s suppression of ASK1, ERK, JNK, and P38 phosphorylation (Figures 9A–E). These findings support the hypothesis that Pac alleviates ferroptosis in MASLD by activating PPARα, which in turn inhibits MAPK signaling. Thus, the Pac-PPARα-MAPK axis mechanistically explains its protective effects against ferroptosis in MASLD.

## 4 Discussion

Metabolic dysfunction-associated steatotic liver disease (MASLD), a globally prevalent chronic liver disorder with risks of progression to hepatocellular carcinoma, faces therapeutic limitations in current strategies. Traditional Chinese Medicine (TCM), notably bioactive compounds like Pac from Poria cocos, offers promise through multifaceted mechanisms. Our study demonstrates that Pac alleviates hepatic steatosis in HFD-induced MASLD mice by enhancing antioxidant defenses—reducing lipid peroxidation markers (LPO, MDA, 4-HNE) and Fe^2+^ accumulation while elevating total hepatic iron content. Pac upregulates ferroptosis suppressors (SLC7A11, GPX4) and downregulates TRF1, concurrently mitigating HFD-induced hepatocyte apoptosis. Transcriptomic analyses suggest Pac’s efficacy involves coordinated modulation of ferroptosis and apoptosis pathways. Compared to YSF (a hepatoprotective agent of Western origin), Pac exhibits comparable efficacy with advantages in dosage precision and mechanistic clarity, bridging traditional holistic approaches with targeted pharmacological intervention. These findings highlight Pac’s potential as a mechanistically defined therapeutic candidate for MASLD, warranting further clinical validation to optimize its integration into liver disease management strategies.

Emerging evidence underscores PPARα′s pivotal role in MASLD, where it regulates lipid metabolism and facilitates mitochondrial fatty acid translocation (Zhong et al., 2023). PPARα activation mitigates iron overload-induced ferroptosis by modulating GPX4 and TRF1 (Xing et al., 2022), while MDM2/MDMX-mediated PPARα regulation induces ferroptosis through lipid remodeling (Venkatesh et al., 2020).

Our transcriptomic analysis identified PPARα as the most significantly altered gene in Pac-treated MASLD mice. Pac upregulated hepatic PPARα protein expression, and the PPARα inhibitor GW6471 reversed Pac’s effects on LPO, MDA, 4-HNE, Fe^2+^, SLC7A11, GPX4, and TRF1 (Supplementary Figure S1E; Figure 8A-E). These findings suggest Pac acts as a PPARα agonist, alleviating ferroptosis and lipid accumulation in MASLD.

Remarkably, Pac may exert anti-MASLD effects by modulating the MAPK signaling pathway, as revealed in our transcriptomic analysis. The phosphorylation equilibrium of MAPKs is central to inflammatory signaling cascades (Li et al., 2023). The MAPK pathway regulates inflammation and oxidative stress via Nrf2 and NF-κB, both linked to hepatic disorders (Ding et al., 2023). Recent studies associate MAPK activation with ferroptosis (Wang et al., 2023), where iron overload and lipid peroxidation alter MAPK dynamics (Chen et al., 2023).

Iron overload impedes MAPK phosphorylation, exacerbating oxidative stress and impairing muscle regeneration in CTX-induced injury (Ikeda et al., 2019). Conversely, excess iron increases p38 and c-FOS phosphorylation, worsening hepatocyte damage (Salama and Kabel, 2020). Taxifolin counteracts iron-induced oxidative stress by inhibiting MAPK signaling.

In parallel, our results revealed that Pac administration markedly represses the phosphorylation of ASK1, P38, ERK1/2, and JNK1/2, suggesting that Pac may mitigate ferroptosis by dampening the MAPK signaling pathway. It is noteworthy that prior research has identified MAPKs as crucial modulators of PPARα activity (Li et al., 2015). In our investigation, we postulate that Pac may suppress the activation of the MAPK pathway by enhancing PPARα activity, thereby alleviating ferroptosis in the livers of MASLD mice. Indeed, the use of the PPARα inhibitor GW6471 significantly reversed the Pac-mediated inhibition of MAPK pathway activation in MASLD mice.

In summary, our collective evidence substantiates the identification of Pac as a potent ligand for PPARα, and delineates its efficacy in alleviating the progression of MASLD. Through its dual mechanism of action, reducing hepatic lipid accumulation and inhibiting ferroptosis, Pac showcases a promising therapeutic potential for the management of MASLD. The elucidation of Pac’s role as a PPARα agonist and its subsequent impact on MASLD pathogenesis opens new avenues for the development of targeted therapies. By addressing the core pathophysiological processes that underpin MASLD, including dysregulated lipid metabolism and cell death pathways, Pac provides a novel approach to counteract the disease’s deleterious effects.

## 5 Conclusion

Our findings contribute to the growing body of knowledge on the molecular mechanisms underlying MASLD and highlight the importance of PPARα activation in the context of liver health. Moreover, they emphasize the potential of natural compounds, such as Pac derived from traditional Chinese medicine, in the design of innovative therapeutic strategies for MASLD. Moving forward, these insights will inform preclinical and clinical studies aimed at validating the efficacy and safety of Pac and other PPARα agonists as therapeutic agents for MASLD. Such investigations will be instrumental in translating our findings into practical applications, potentially revolutionizing the treatment landscape for this prevalent and challenging liver disorder.

## Data Availability

The original contributions presented in the study are publicly available. This data can be found here: http://www.ncbi.nlm.nih.gov/bioproject/1257833.

## Glossary

ALT: Alanine aminotransferase
AMPK: Adenosine monophosphate-activated protein kinase
AST: Aspartate aminotransferase
BCA: Bicinchoninic acid
BSA: Bovine serum albumin
CD36: Scavenge receptor B2
CPT1α: Carnitine palmitoylransferase 1α
ERK: Mitogen-activated protein kinase 1
FASN: Fatty acid synthase
FATP5: solute carrier family 27 member 5
GPX4: Glutathione peroxidase 4
GW6471: PPARα inhibitor
H&E: Hematoxylin-Eosin staining
JNK: Mitogen-activated protein kinase 8
LPO: Lipid hydroperoxides
MAPKs: Mitogen-activated protein kinases
MDA: Malondialdehyde
MASL: Metabolic dysfunction-associated steatotic liver
MASLD: Metabolic dysfunction-associated steatotic liver disease
MASH: Metabolic dysfunction-associated steatohepatitis
OA: Sodium oleate
PA: Sodium palmitate
Pac: Pachymaric acid
P-AMPK: Phosphorylated adenosine monophosphate-activated protein kinase
P-ERK: Phosphorylated mitogen-activated protein kinase 1
P-JNK: Phosphorylated mitogen-activated protein kinase 8
PO: BSA-Palmitate Saturated Fatty Acid Complex
BSA-Oleate: monounsaturated fatty acid complex(2:1)
PPARα: Peroxisome proliferator activated receptor alpha
PPARs: (Peroxisome proliferators-activated receptors)
P-P38: Phosphorylated mitogen-activated protein kinase 14
P38: Mitogen-activated protein kinase 14
SCD1: Stearoyl cenzyme A desaturase
SDS-PAGE: Sodium dodecyl sulfate - Polyacrylamide gel electrophoresis
SLC7A11: Solute carrier family 7 member 11
SREBP1c: Sterol regulatory element-binding protein 1c
TC: Total cholesterol; TFR1, transferrin receptor
TG: Triglyceride
VLDLR: Very low-density lipoprotein receptor;
β-actin: Beta-actin
4-HNE: 4-Hydroxy-nonenal
