# Supplementary material for: Pachymic acid alleviates metabolic dysfunction-associated steatotic liver disease by inhibiting ferroptosis through PPARα
Source: Front Pharmacol. 2025 May 6;16:1554850. doi: 10.3389/fphar.2025.1554850 (PMC12088974; doi:10.3389/fphar.2025.1554850)
Supplement: Supplementary file 4 [file DataSheet1.doc]

**Pachymic Acid Alleviates Non-Alcoholic Fatty Liver Disease by Inhibiting Ferroptosis through Activation of PPARα**

Guilin Ren^a,b†^, Yiyou Lin^a,b†^, Jiannan Qiu^a,b^, Congcong Zhang^a,b^, Lin Chen^a,b^, Linwensi Zhu^c^, Xiaohui Fan^d^, Xiaobing Dou^a,b^*, Qingsheng Liu^e^*

a School of Life Science, Zhejiang Chinese Medical University, Hangzhou, Zhejiang, PR China. 310053

b Zhejiang-Hong Kong Joint Laboratory of Liver and Spleen Simultaneous Treatment in Traditional Chinese Medicine, Zhejiang, PR China. 310053

c The First Affiliated Hospital of Zhejiang Chinese Medical University, Hangzhou, Zhejiang, PR China. 310006

d Innovation Center of Yangtze River Delta, Zhejiang University, Jiaxing, Zhejiang, PR China. 314100

e Hangzhou Third People's Hospital, Hangzhou, Zhejiang, PR China. 310009

* Correspondence author:

Xiaobing Dou, School of Life Science, Zhejiang Chinese Medical University, Hangzhou, Zhejiang, PR China; E-mail addresses: xbdou77@gmail.com

Qingsheng Liu, Hangzhou Third People's Hospital, Hangzhou, Zhejiang, PR China. Email addresses: 7394822@qq.com

† These authors contributed equally to this work.


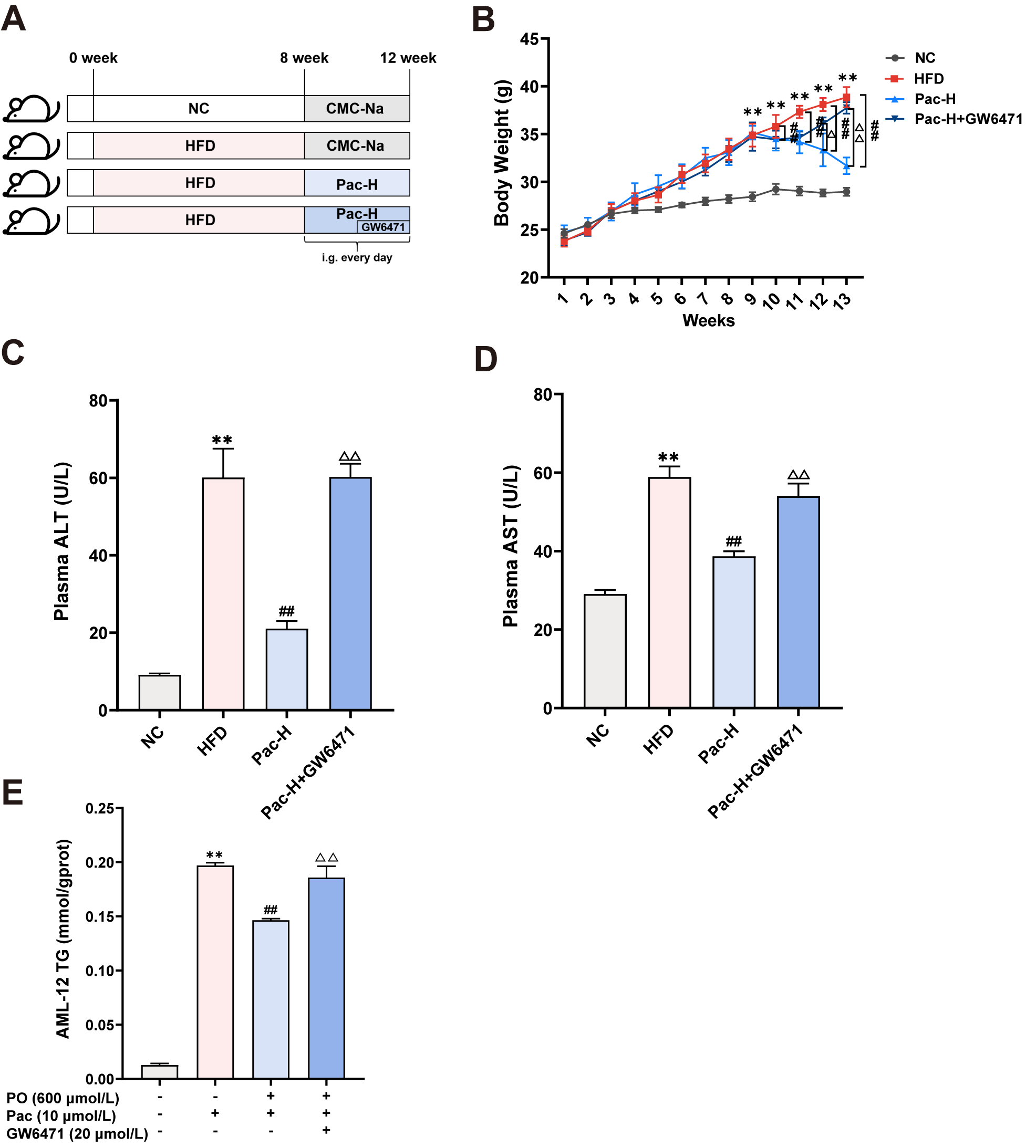


Figure S1. Pac effectively reduced body weight and injury in NAFLD mice.

(A) Experimental flowchart. (B) The wight growth curve of each group (weekly). (C, D) The level of plasma ALT and AST. (E) The level of AML-12 TG (n=6). Data are showed as mean ± SD (n = 8). **p* value for v.s. NC < 0.05, ***p* value for v.s. NC < 0.01, **^#^**p value for v.s. HFD < 0.05,**^##^**p value for v.s. HFD < 0.01, **^△^**p value for v.s. Pac < 0.05, **^△△^**p value for v.s. Pac < 0.01.
