# Supplementary material for: Pachymic acid alleviates metabolic dysfunction-associated steatotic liver disease by inhibiting ferroptosis through PPARα
Source: Front Pharmacol. 2025 May 6;16:1554850. doi: 10.3389/fphar.2025.1554850 (PMC12088974; doi:10.3389/fphar.2025.1554850)
Supplement: Supplementary file 8 [file DataSheet2.docx]

**Original Western blots**

**Figure. 3**

| CD36 88 kDa | 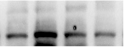  88 kDa | 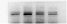  88 kDa | 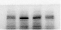  88 kDa |
| --- | --- | --- | --- |
| VLDLR 96 kDa | 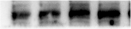  96 kDa | 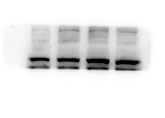  96 kDa | 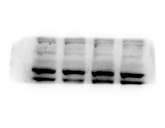  96 kDa |
| FATP5 75kDa | 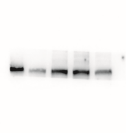  75 kDa | 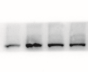  75 kDa | 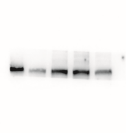  75 kDa |
| SREBP1c 125 kDa | 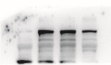  125 kDa | 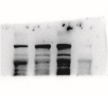  125 kDa | 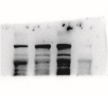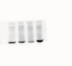  125 kDa |
| FASN 273 kDa | 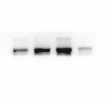  273 kDa | 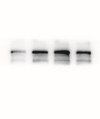  273 kDa | 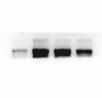  273 kDa |
| SCD1  37 kDa | 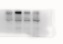  37 kDa | 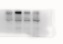  37 kDa | 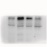  37 kDa |
| p-AMPK 62 kDa | 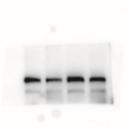  62 kDa | 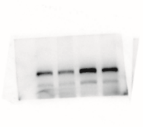  62 kDa | 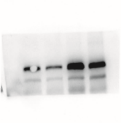  62 kDa |
| AMPK 62 kDa | 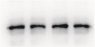  62 kDa | 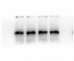  62 kDa | 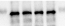  62 kDa |
| CPT1α  88 kDa | 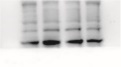  88 kDa | 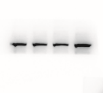  88 kDa | 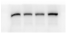  88 kDa |
| PPARα  55 kDa | 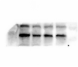  55 kDa | 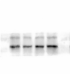  55 kDa | 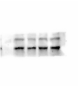  55 kDa |
| β-actin  42 kDa | 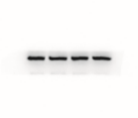  42 kDa | 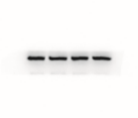  42 kDa | 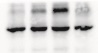  42 kDa |

**Figure. 4**

| 4-HNE | 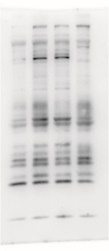 | 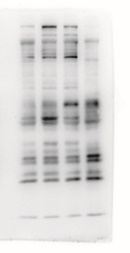 | 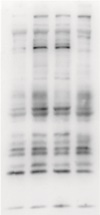 |
| --- | --- | --- | --- |
| β-actin  42 kDa | 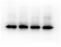  42 kDa | 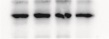  42 kDa | 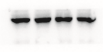  42 kDa |
| GPX4  17 kDa | 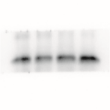  17 kDa | 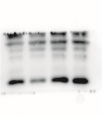  17 kDa | 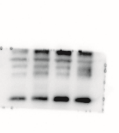  17 kDa |
| SLC7A11  63 kDa | 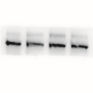  63kDa | 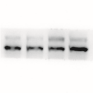  63 kDa | 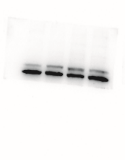  63 kDa |
| β-actin  42 kDa | 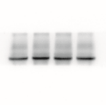  42 kDa | 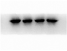  42 kDa | 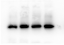  42 kDa |

**Figure. 5**

44/42 kDa

| p-ASK1  155 kDa | 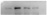  155 kDa | 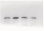 | 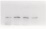 |
| --- | --- | --- | --- |
| ASK1  155 kDa | 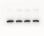  155 kDa | 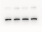  155 kDa  155 kDa | 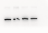  155 kDa  155 kDa |
| p-ERK  44/42 kDa | 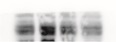  44/42 kDa | 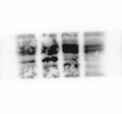 | 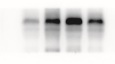  44/42 kDa |
| ERK  44/42 kDa | 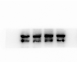  44/42 kDa | 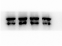  44/42 kDa | 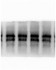  44/42 kDa |
| p-JNK  54/46 kDa | 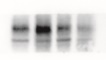  54/46 kDa | 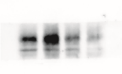  54/46 kDa | 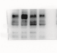  54/46 kDa |
| JNK  54/46 kDa | 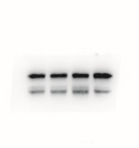  54/46 kDa | 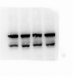  54/46 kDa | 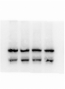  54/46 kDa |
| p-P38  38 kDa | 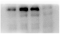 | 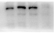  38 kDa | 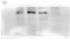  38 kDa  38 kDa |
| P38  38 kDa | 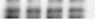  38 kDa | 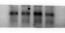  38 kDa | 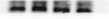  38 kDa |
| β-actin  42 kDa | 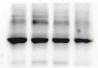  42 kDa | 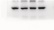  42 kDa | 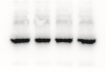  42 kDa |

**Figure. 7**

| CD36 88 kDa | 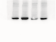  88 kDa | 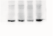  88 kDa | 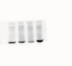  88 kDa |
| --- | --- | --- | --- |
| VLDLR 96 kDa | 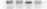  96 kDa | 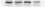  96 kDa | 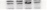  96 kDa |
| FATP5 75kDa | 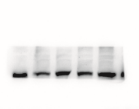  75 kDa | 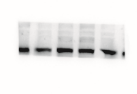  75 kDa | 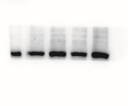  75 kDa |
| SREBP1c 125 kDa | 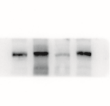  125 kDa | 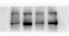  125 kDa | 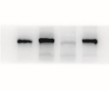  125 kDa |
| FASN 273 kDa | 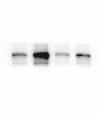  273 kDa | 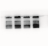  273 kDa  37 kDa | 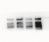  273 kDa |
| SCD1  37 kDa | 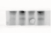  37 kDa | 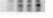 | 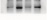  37 kDa |
| p-AMPK 62 kDa | 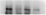  62 kDa | 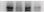  62 kDa | 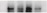  62 kDa |
| AMPK 62 kDa | 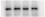  62 kDa | 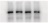  62 kDa | 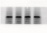  62 kDa |
| CPT1α  88 kDa | 88 kDa | 88 kDa | 88 kDa |
| PPARα  55 kDa | 55 kDa | 55 kDa | 55 kDa |
| β-actin  42 kDa | 42 kDa | 42 kDa | 42 kDa |

**Figure. 8**

| 4-HNE |  |  |  |
| --- | --- | --- | --- |
| β-actin  42 kDa | 42 kDa | 42 kDa | 42 kDa |
| GPX4  17 kDa | 17 kDa | 17 kDa | 17 kDa |
| SLC7A11  63 kDa | 63kDa | 63 kDa | 63 kDa |
| β-actin  42 kDa | 42 kDa | 42 kDa | 42 kDa |

**Figure. 9**

38 kDa

54/46 kDa

54/46 kDa

44/42 kDa

| p-ASK1  155 kDa | 155 kDa |  | 155 kDa  155 kDa |
| --- | --- | --- | --- |
| ASK  155 kDa | 44/42 kDa  155 kDa | 155 kDa | 155 kDa |
| p-ERK  44/42 kDa |  | 44/42 kDa |  |
| ERK  44/42 kDa | 44/42 kDa |  | 44/42 kDa  44/42 kDa |
| p-JNK  54/46 kDa | 54/46 kDa | 54/46 kDa |  |
| JNK  54/46 kDa | 54/46 kDa |  | 54/46 kDa |
| p-P38  38 kDa | 38 kDa |  | 38 kDa  38 kDa |
| P38  38 kDa | 38 kDa |  | 38 kDa |
| β-actin  42 kDa | 42 kDa | 42 kDa | 42 kDa |
